# Remote Communication and Loneliness During the COVID-19 Pandemic: Cross-Sectional Study

**DOI:** 10.2196/45338

**Published:** 2023-07-11

**Authors:** Yuki Arakawa, Kosuke Inoue, Daisuke Nishioka, Atsushi Nakagomi, Takahiro Tabuchi, Naoki Kondo

**Affiliations:** 1 Department of Social Medicine Graduate School of Medicine The University of Tokyo Tokyo Japan; 2 Department of Social Epidemiology Graduate School of Medicine Kyoto University Kyoto Japan; 3 Department of Medical Statistics Research & Development Center Osaka Medical and Pharmaceutical University Osaka Japan; 4 Department of Social Preventive Medical Sciences Center for Preventive Medical Sciences Chiba University Chiba Japan; 5 Cancer Control Center Osaka International Cancer Institute Osaka Japan

**Keywords:** loneliness, remote communication, social isolation, information and communications technology, ICT, COVID-19, restrictions, communication tool, age, gender, text message

## Abstract

**Background:**

Although remote communication technologies have been widely used to maintain connections with others against interpersonal contact restrictions and exacerbated loneliness during the COVID-19 pandemic, it is unclear whether and what types of remote communication technologies are effective in mitigating loneliness.

**Objective:**

This study aimed to investigate the association between remote communication and loneliness when face-to-face meetings with others were strongly prohibited and whether this association varied across types of communication tools, age, and gender.

**Methods:**

We used cross-sectional data from the Japan COVID-19 and Society Internet Survey conducted from August to September 2020. From registered panelists of the research agency, 28,000 randomly sampled participants completed the survey on the website. We created 2 study cohorts who stopped meeting with family members living apart and friends during the pandemic. We categorized whether participants had technology-based remote communication (voice calling, text messaging, and video calling) with family and friends. Loneliness was assessed using the 3-item University of California, Los Angeles Loneliness Scale. We used a modified Poisson regression model to investigate the association between loneliness and remote communication with family members living apart or friends. We also conducted subgroup analyses based on age and gender.

**Results:**

A total of 4483 participants stopped meeting with family members living apart, and 6783 participants stopped meeting with friends during the COVID-19 pandemic. Remote communication with family members living apart did not show an association with loneliness, whereas remote communication with friends was associated with a low prevalence of loneliness (family: adjusted prevalence ratio [aPR]=0.89, 95% CI 0.74-1.08; *P*=.24 and friends: aPR=0.82, 95% CI 0.73-0.91; *P*<.001). From analyses by tools, voice calling was associated with low loneliness (family: aPR=0.88, 95% CI 0.78-0.98; *P*=.03 and friends: aPR=0.87, 95% CI 0.80-0.95; *P*=.003). Similarly, text messaging was associated with low loneliness (family: aPR=0.82, 95% CI 0.69-0.97; *P*=.02 and friends: aPR=0.81, 95% CI 0.73-0.89; *P*<.001). However, we did not find an association between video calling and loneliness (family: aPR=0.88, 95% CI 0.75-1.02; *P*=.09 and friends: aPR=0.94, 95% CI 0.85-1.04; *P*=.25). Text messaging with friends was associated with low loneliness regardless of age, whereas voice calling with family or friends was associated with low loneliness only among participants aged ≥65 years. An association between remote communication with friends and low loneliness was found regardless of the type of remote communication tool among men, whereas it was found only for text messaging with friends among women.

**Conclusions:**

In this cross-sectional study of adults in Japan, remote communication, especially via voice calling and text messaging, was associated with low loneliness. Promoting remote communication may reduce loneliness when face-to-face contact is restricted, which should be the subject of future research.

## Introduction

Loneliness is a subjective negative experience characterized by a discrepancy between an individual’s desired and actual social relationships [1]. Loneliness has become a public health concern because it predicts negative health status, including depression [2], suicide attempts [3], cardiovascular disease [4], and mortality [5]. Moreover, the COVID-19 pandemic has exacerbated loneliness worldwide [6,7]. This is partly because of infection control policies that have been implemented to curb the spread of the virus, such as social distancing, limiting gatherings, and reducing face-to-face contact with others [8-10]. In this context, it is imperative to establish effective countermeasures to maintain social relationships in mitigating loneliness during this unprecedented time.

The sense of being connected to close ones, such as their spouse, family, or friends, is a critical factor against loneliness, even at a distance [11]. The recent development of information and communication technologies, including chat apps and video calling, has enabled more convenient, constant, and visualized remote communication. This technology-based remote communication may be useful for creating a sense of connectedness, even if people have not met in person [12,13]. Indeed, being on the web and social media communication with family or friends were related to less loneliness among the older adult population [14,15]. In contrast, studies during the COVID-19 pandemic and a recent systematic review have suggested that the effect of technology-based remote communication in alleviating loneliness is uncertain [16-18]. The mixed findings may be attributable to the difference in face-to-face contact frequencies or the type of communication tools used across studies.

Furthermore, the impact of technology-based remote communication on loneliness may vary according to sociodemographic factors such as age and gender. The association between the frequency of social contact and loneliness was stronger in younger adults than in older adults [19]. However, the association between not working and loneliness compared with working full time was found only in middle-aged adults [20]. As daily technology use differs across age groups, the impact of remote communication on loneliness during a pandemic can also vary. Regarding gender, women are more likely to maintain relationships using text messaging or share information through the internet than men, which may yield a gender difference in the impact of remote communication on loneliness [21,22].

The effects of remote communication on loneliness could depend on whether face-to-face communication is effortless. However, to our knowledge, no study has evaluated the impact of technology-based remote communication with family or friends on reducing loneliness when face-to-face interactions are strongly restricted, such as during the COVID-19 pandemic. Furthermore, the age- and gender-specific effects of remote communication on loneliness have not been adequately evaluated. To address these gaps, we investigated whether technology-based remote communication with family members living apart or friends was associated with a low prevalence of loneliness among people who stopped meeting them during the COVID-19 pandemic. We also explored whether the association varied across communication tools and among different age and gender groups.

## Methods

### Data Sources

We used data from the Japan COVID-19 and Society Internet Survey (JACSIS) conducted in 2020. The JACSIS 2020 is a cross-sectional, internet-based, and self-reported questionnaire survey administered by an internet research agency with 2.2 million qualified panelists in Japan (Rakuten Insight). The survey panel was recruited through services managed by the research agency group. The inclusion criteria of the survey panel were people who agreed to (1) provide their information, including sex, age, occupation, and residence, and (2) participate in different research surveys in the future. The panelists comprised individuals from diverse socioeconomic backgrounds, such as household income, educational level, and marital status.

The research agency randomly sampled 224,389 people from this panel stratified by age, gender, and living prefecture to request participation in the JACSIS 2020 survey by email. People who received participation requests accessed the survey website and completed the questionnaire. The questionnaire included various socioeconomic, lifestyle, and health factors. Only people who completed the survey were enrolled for the study. The survey enrollment continued until the predefined target number of participants in terms of age, gender, and prefectures was reached based on the distribution of the general Japanese population in 2019. The survey was conducted between August 25 and September 30, 2020. Consequently, 28,000 participants answered the survey, with an overall response rate of 12.48% (28,000/224,389).

### Study Population

Of the 28,000 participants, we excluded 2518 (8.99%) artificial or unnatural responses to validate the data quality with the following criteria: (1) participants selected invalid responses to “Please choose the option second from the bottom,” (2) participants answered using “all” substance or drugs (ie, sleeping pills, opioids, cocaine, etc), and (3) participants answered having “all” chronic diseases (eg, diabetes, asthma, stroke, and ischemic heart disease). We then included participants aged ≥20 years to focus on independent adults (participants aged <20 years were excluded; n=1214). In addition, we excluded participants who answered that there were >10 household members because their answers were not equal to the number of household members calculated by other questions (n=66); we excluded participants who selected their educational level as “others” (n=59) because this category included few participants, and we could not categorize them by educational attainment years.

We created the following two study cohorts: (1) people who had face-to-face contact with family members living apart before the COVID-19 pandemic but not during the pandemic and (2) people who had face-to-face contact with friends before the COVID-19 pandemic but not during the pandemic. We labeled cohort 1 as the cohort with family members and cohort 2 as the cohort with friends. In our survey, we assessed the frequency of face-to-face communication with family members living apart or with friends before January 2020 and in the past month before August 2020 (before and during the COVID-19 pandemic). The participants answered the frequency of face-to-face communication within the following seven options: (1) “not at all,” (2) “once a month,” (3) “2-3 times a month,” (4) “once a week,” (5) “2-3 times a week,” (6) “4-5 times a week,” and (7) “almost every day (6-7 times a week).” Detailed questionnaires are provided in Table S1 in Multimedia Appendix 1. To create the cohort with family members, we defined people who had face-to-face contact with family members living apart before the COVID-19 pandemic as answering the frequency more than “once a month” (from option 2 to 7) before January 2020. We also defined people who did not have face-to-face contact with their family members living apart during the COVID-19 pandemic as answering that frequency “not at all” in the past month in August 2020. We applied the same approach to create the cohort with friends by the answers regarding the frequency of face-to-face communication with friends.

### Exposure Variables

We defined technology-based remote communication as using one of the following three types of communication: (1) voice calling, (2) text messaging, and (3) video calling. In this survey, we assessed the frequency of each type of remote communication with family members living apart and friends before and during the COVID-19 pandemic (before January 2020 and from August to September 2020; Table S1 in Multimedia Appendix 1). First, we confirmed three questions for each participant in the cohort with family members: (1) voice calling, (2) text messaging, and (3) video calling with family members living apart, defined by using each type of remote communication once a month or more during the pandemic as having communication. We categorized the study participants as having technology-based remote communication if they answered using any of the 3 types of remote communication with family members living apart during the pandemic. Similarly, we assessed the cohort with friends as having each type of remote communication with friends and categorized them as having technology-based remote communication with them or not in the same manner.

### Outcome Variables

Loneliness at the time of the survey was the outcome variable. We used the 3-item version of the University of California, Los Angeles (UCLA) Loneliness Scale (3-item UCLA Loneliness Scale) to assess loneliness [23]; the Japanese version of the 3-item UCLA Loneliness Scale was previously validated [24,25]. The items were as follows: (1) “How often do you feel you lack companionship?” (2) “How often do you feel left out?” and (3) “How often do you feel isolated from others?” Participants selected the frequency with which these feelings were experienced over the past 30 days using 5 options ranging from 1 to 5 (1=never, 2=rarely, 3=sometimes, 4=often, and 5=always). These options were modified from the original 3 options of the 3-item UCLA Loneliness Scale (1=hardly ever, 2=some of the time, and 3=often) because we had to modify the response options of the UCLA Loneliness Scale to align with the measured scale (Kessler Psychological Distress Scale [K6]) based on a 5-point Likert scale. The total 3-item UCLA Loneliness Scale score in the original version ranged from 3 to 9. Recent studies have used the cutoff point to define the lonely state [26,27]. To use this cutoff point, we matched our scale’s answer options to the original one so that the scores would be equivalent. In our survey, therefore, we reassigned a score of 1 point for the participants selecting “never” or “rarely,” 2 points for selecting “sometimes,” and 3 points for selecting “often” or “always.” Therefore, we summed up the total points of 3 items, which ranged from 3 to 9, similar to the original version, with a higher score indicating greater loneliness. As used in recent studies, we defined a lonely state as those with a summed score of ≥5 points [26,27].

### Adjustment Variables

We adjusted for the participants’ sociodemographic factors, past chronic diseases, past mental problems, frequency of face-to-face contact with family members living apart or friends before the COVID-19 pandemic, and previous use of exposure variables before the COVID-19 pandemic. The sociodemographic factors included age (range 20-79 years), gender (man or woman), marital status (married, never married, divorced, or widowed), household size (number of household members), educational level (graduated from college or institutions of higher education vs high school or lower institutions), household income level categorized by the tertile of household equivalent income (“low,” <¥2.5 million Japanese yen; “medium,” ¥2.5-4.3 million Japanese yen; “high,” >¥4.3 million Japanese yen; “unknown”; and “declined to answer”; a currency exchange rate of ¥1 yen=US $0.0071 is applicable), and working status (having any work, full-time housekeeping, not working, or student). The equivalent income level was calculated by dividing the household income by the square root of the number of household members. We categorized participants into “having past chronic diseases” if participants had any 1 of the following diseases: hypertension, diabetes mellitus, asthma, angina, myocardial infarction, stroke, chronic obstructive pulmonary disease, and cancer. We also categorized participants into “having past mental problems” if they had depression or other mental illnesses. We adjusted for the frequency of face-to-face communication (“once a month,” “2-3 times a month,” “once a week,” or “2-3 times a week or more”) with family members living apart before January 2020 for the first cohort and with friends before January 2020 for the second cohort. We also adjusted for the previous use of remote communication tools before the COVID-19 pandemic.

### Statistical Analysis

We used a 2-level modified Poisson regression model to estimate the adjusted prevalence ratio (aPR) of loneliness among the cohort with family members and the cohort with friends [28]. We considered each participant as the first level and the prefectures each participant lived in (n=47) as the second level in this multilevel analysis. We included all the abovementioned adjustment variables with squared age in the first level. For the second level, our study participants were drawn from all 47 prefectures in Japan, and each study sample lived in 1 of the 47 prefectures. Many factors, such as the spread of infection, preventive measures against COVID-19, urbanity, transportation, culture, and information and communications technology penetration rate by generation, were shared among the same prefectures and differed across other prefectures. Thus, we adopted multilevel analysis and considered the prefectures each participant lived in as the second-level variables. All analyses were conducted using Stata software (version 16.0; StataCorp LLC), including the *mepoisson* command. We incorporated random intercepts at the prefecture level. We conducted subgroup analyses using the same model by age group (aged >65 years and ≥65 years) and gender. To discover the between-group difference, we first estimated the point estimate of aPR for loneliness with 95% CI in each subgroup and calculated *P* value for interactions using the Altman method [29]. We used 65 years as a cutoff for age groups based on previous studies and reviews [30,31], as most Japanese people retire at age 65 years [32], which can yield lifestyle differences between the groups, such as communication frequencies, communication partners, and how they use technology-based communication tools. In addition, we used 60 years as a cutoff for sensitivity analysis.

### Ethics Approval, Informed Consent, and Participant Privacy

The study was reviewed and approved by the Research Ethics Committee of the Osaka International Cancer Institute (20084). All the respondents provided informed consent to participate in the study. Participants’ privacy was protected because all data were anonymous.

## Results

**Figure 1:**
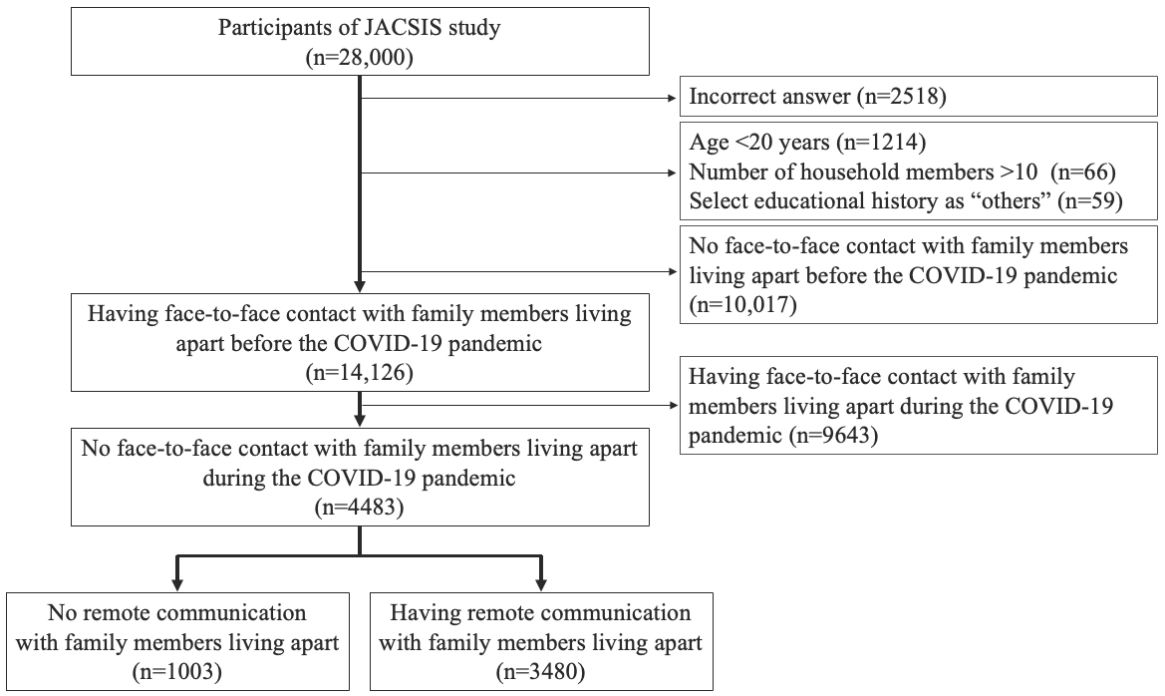
Selection flowchart of study participants who had face-to-face contact with family members living apart before but not during the COVID-19 pandemic (cohort 1). JACSIS: Japan COVID-19 and Society Internet Survey.

We included 4483 study participants who had face-to-face contact with family members living apart before the COVID-19 pandemic but not during the pandemic in the cohort with family members (Figure 1). Among this cohort, 77.63% (3480/4483) of the participants had remote communication with family members living apart during the pandemic.

**Figure 2:**
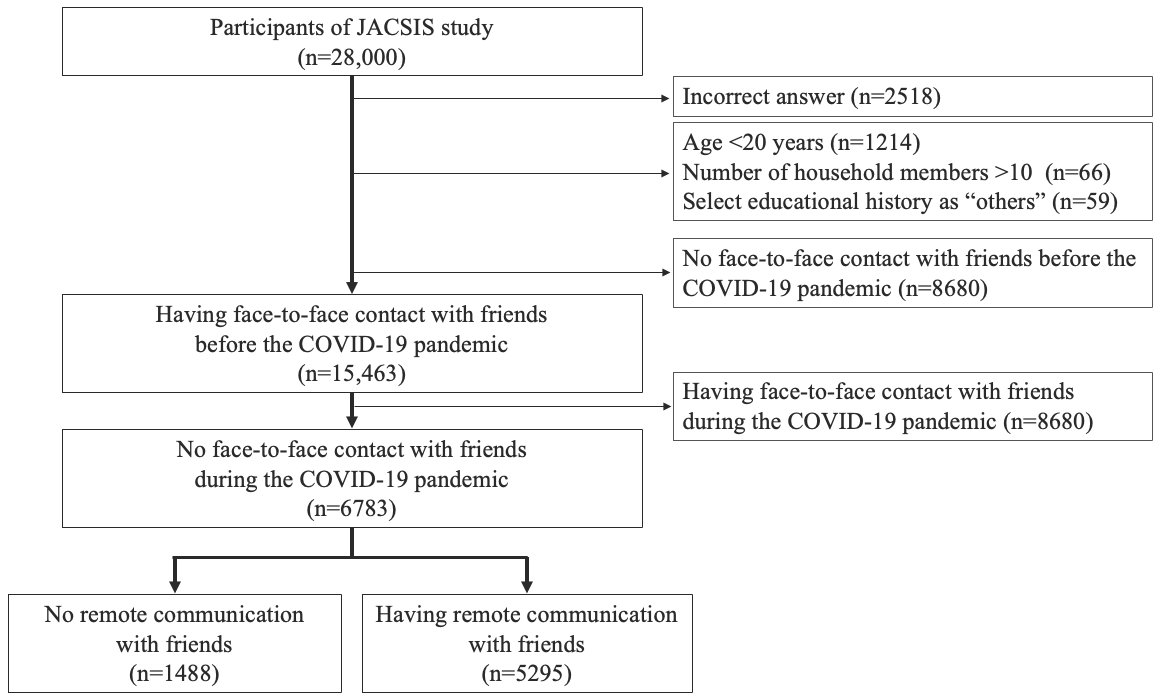
Selection flowchart of study participants who had face-to-face contact with friends before but not during the COVID-19 pandemic (cohort 2). JACSIS: Japan COVID-19 and Society Internet Survey.

We included 6783 study participants who had face-to-face contact with friends before the COVID-19 pandemic but not during the pandemic in the cohort with friends (Figure 2). Among this cohort, 78.06% (5295/6783) of the participants had remote communication with friends during the pandemic.

The characteristic differences between the study cohort and the people who had face-to-face contact with family members living apart or friends during the COVID-19 pandemic in the JACSIS 2020 study are shown in Table S2 in Multimedia Appendix 1. The mean age of the cohort with family members and the cohort with friends was 52.3 (SD 16.2) years and 52.2 (SD 15.7) years, respectively. The median age in each cohort was 53 years. Across each study group in the 2 cohorts, the mean age ranged from 51.5 to 54 years, and the distribution was similar (Table 1 and Table 2).

Participants who had remote communication with family members living apart or with friends were mostly women, had higher income, had higher educational level, were married, were housekeeping, and were living alone. In addition, more participants who had remote communication with family members living apart were married in the cohort with family members. Approximately half (cohort 1, 441/1003, 43.97%; cohort 2, 772/1488, 51.88%) of the study participants who had remote communication with family members living apart or with friends before the pandemic did not have remote communication with them during the pandemic.

**Figure 3:**
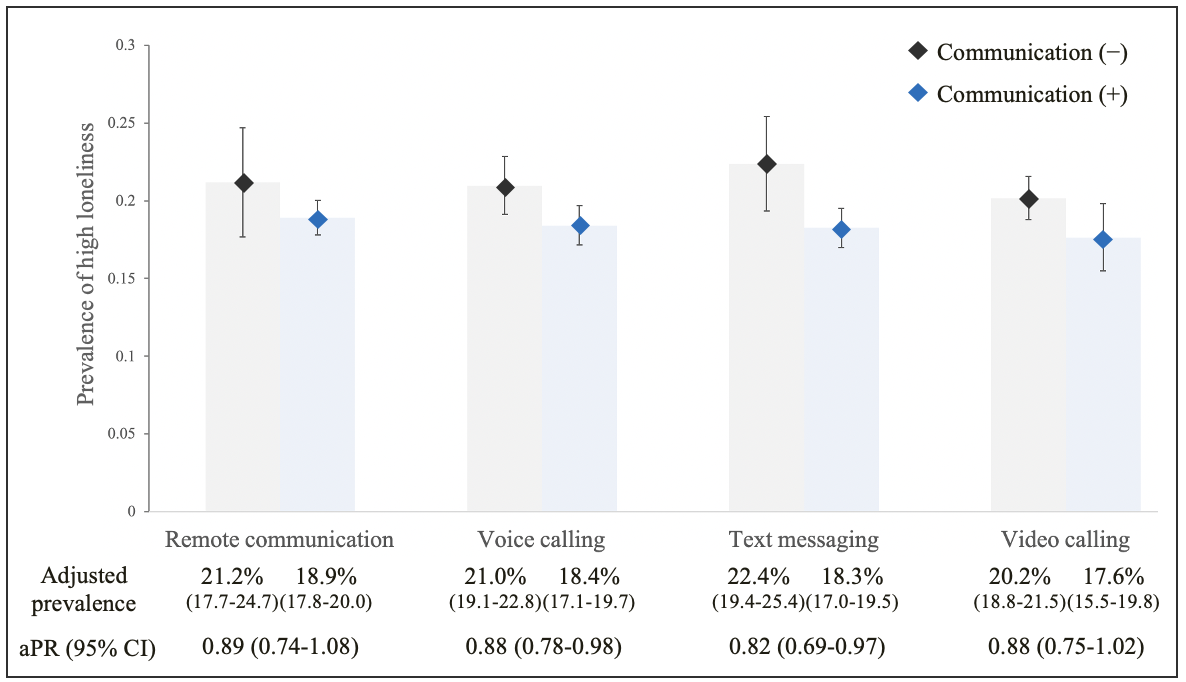
Association between remote communication with family members living apart and loneliness among people who stopped meeting with them during the COVID-19 pandemic. Adjusted for age (singular and squared terms), gender, income, education level, work status, marital status, number of household members, chronic disease, past mental problem, frequency of face-to-face contact before the COVID-19 pandemic, and frequency of exposure variables before the COVID-19 pandemic. aPR: adjusted prevalence ratio.

Having remote communication with family members living apart did not show an apparent association with a low prevalence of loneliness, although the point estimate of aPR was 0.89 (95% CI 0.74-1.08; *P*=.24; Figure 3).

**Figure 4:**
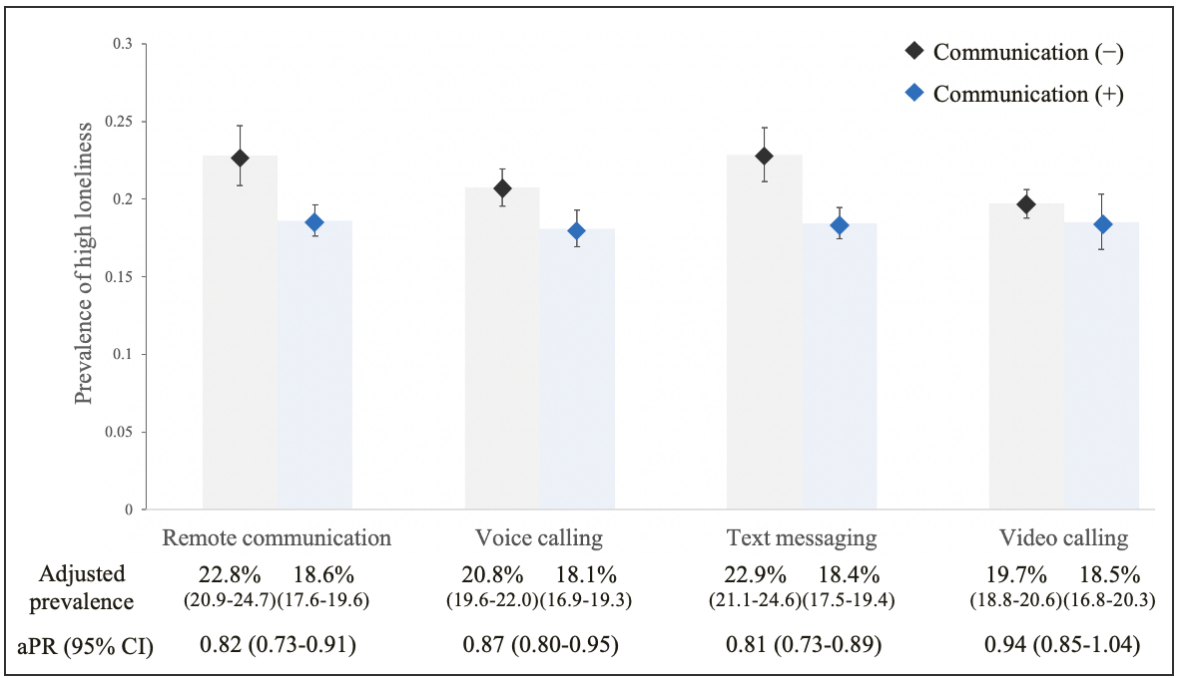
Association between remote communication with friends and loneliness among people who stopped meeting with them during the COVID-19 pandemic. Adjusted for age (singular and squared terms), gender, income, education level, work status, marital status, number of household members, chronic disease, past mental problem, frequency of face-to-face contact before the COVID-19 pandemic, and frequency of exposure variables before the COVID-19 pandemic. aPR: adjusted prevalence ratio.

In contrast, having remote communication with friends was associated with a low prevalence of loneliness (aPR=0.82, 95% CI 0.73-0.91; *P*<.001; Figure 4).

We found that the point estimate of the aPR of loneliness was smaller among the participants having remote communication with friends than that with family members. Our analyses by tool showed that having a voice call with family members living apart or friends was associated with a low prevalence of loneliness (family: aPR=0.88, 95% CI 0.78-0.98; *P*=.03 and friends: aPR=0.87, 95% CI 0.80-0.95; *P*=.003). Similarly, engaging in text messaging with family members living apart or friends was associated with a low prevalence of loneliness, and the association was stronger than that of voice calling with them (family: aPR=0.82, 95% CI 0.69-0.97; *P*=.02 and friends: aPR=0.81, 95% CI 0.73-0.89; *P*<.001). Having a video call with family members living apart or friends did not show clear associations with loneliness (family: aPR=0.88, 95% CI; 0.75-1.02; *P*=.09 and friends: aPR=0.94, 95% CI 0.85-1.04; *P*=.25).

There was no evidence of heterogeneity by age group (age range: <65 years, 20-64 years, ≥65 years, and 65-79 years) in the association between having remote communication and loneliness (Table 3).

**Table 1:** Sociodemographic and communication characteristics in the study population who stopped meeting family members living apart during the COVID-19 pandemic.

Characteristics				No face-to-face contact with family members living apart during the pandemic (Cohort 1, n=4483)		
				No remote communication with family members living apart (n=1003)		Having remote communication with family members living apart (n=3480)
**Demographic factors**						
	**Age (years)**					
		Value, mean (SD)	51.5 (15.9)		52.5 (16.2)	
		Value, median (range)	52 (20-79)		54 (20-79)	
	**Gender, n (%)**					
		Man	607 (60.52)		1462 (42.01)	
		Woman	396 (39.48)		2018 (57.99)	
	**Income (¥; ¥1 yen=US $0.0071), n (%)**					
		Low (<250 million)	309 (30.81)		844 (24.25)	
		Intermediate (250-430 million)	256 (25.52)		980 (28.16)	
		High (>430 million)	216 (21.53)		1029 (29.57)	
		Refusal to answer	127 (12.66)		343 (9.86)	
		Unknown	95 (9.47)		284 (8.16)	
	**Education, n (%)**					
		≤12 years	312 (31.11)		872 (25.06)	
		>12 years	691 (68.89)		2608 (74.94)	
	**Employment status, n (%)**					
		Working	650 (64.81)		2034 (58.45)	
		House keeping	137 (13.66)		800 (22.99)	
		Not working	185 (18.44)		542 (15.57)	
		Student	31 (30.09)		104 (2.99)	
	**Marital status, n (%)**					
		Married	631 (62.91)		2466 (70.86)	
		Single	268 (26.72)		670 (19.25)	
		Widowed or divorced	104 (10.37)		344 (9.88)	
	**Household size, n (%)**					
		1	152 (15.15)		619 (17.79)	
		2	364 (36.29)		1392 (40)	
		>3	487 (48.55)		1469 (42.21)	
**Health-related factors, n (%)**						
	Having chronic disease history			422 (42.07)		1424 (40.92)
	Having past mental problem			111 (11.07)		395 (11.35)
**Frequency of face-to-face contact with family members living apart before the pandemic, n (%)**						
	Once a month			722 (71.98)		2664 (76.55)
	2-3 times a month			132 (13.16)		465 (13.36)
	Once a week			76 (7.58)		190 (5.46)
	≥2 times a week			73 (7.28)		161 (4.63)
**Remote communication with family members living apart during the pandemic, n (%)**						
	**Having remote communication**			0 (0)		3480 (100)
		Voice calling^a^	0 (0)		2752 (79.08)	
		Text messaging^a^	0 (0)		3150 (90.52)	
		Video calling^a^	0 (0)		1170 (33.62)	
**Remote communication with family members living apart before the pandemic, n (%)**						
	**Having remote communication**			441 (43.97)		3395 (97.56)
		Voice calling^a^	305 (30.41)		2859 (82.15)	
		Text messaging^a^	324 (32.3)		3125 (89.8)	
		Video calling^a^	111 (11.07)		1001 (28.76)	
Loneliness, n (%)				225 (22.43)		647 (18.59)

^a^We defined people who used each remote communication method once a month or more as using each type of communication.

**Table 2:** Sociodemographic and communication characteristics in the study population who stopped meeting friends during the COVID-19 pandemic.

Characteristics				No face-to-face contact with friends during the pandemic (Cohort 2, n=6783)		
				No remote communication with friends (n=1488)		Having remote communication with friends (n=5295)
**Demographic factors**						
	**Age (years)**					
		Value, mean (SD)	54.0 (15.5)		51.7 (15.7)	
		Value, median (range)	55 (20-79)		52 (20-79)	
	**Gender, n (%)**					
		Man	854 (57.39)		2215 (41.83)	
		Woman	634 (42.61)		3080 (58.17)	
	**Income (¥; ¥1 yen=US $0.0071), n (%)**					
		Low (<250 million)	428 (28.76)		1238 (23.38)	
		Intermediate (250-430 million)	381 (25.6)		1501 (28.35)	
		High (>430 million)	329 (22.11)		1592 (30.07)	
		Refusal to answer	195 (13.1)		517 (9.76)	
		Unknown	155 (10.42)		447 (8.44)	
	**Education, n (%)**					
		≤12 years	471 (31.65)		1263 (23.85)	
		>12 years	1017 (68.35)		4032 (76.15)	
	**Employment status, n (%)**					
		Working	915 (61.49)		3284 (62.02)	
		House keeping	253 (17)		1123 (21.21)	
		Not working	298 (20.03)		773 (14.6)	
		Student	22 (1.48)		115 (2.17)	
	**Marital status, n (%)**					
		Married	1047 (70.36)		3647 (68.88)	
		Single	319 (21.44)		1130 (21.34)	
		Widowed or divorced	122 (8.2)		518 (9.78)	
	**Household size, n (%)**					
		1	207 (13.91)		908 (17.15)	
		2	609 (40.93)		2029 (38.32)	
		>3	672 (45.16)		2358 (44.53)	
**Health-related factors, n (%)**						
	Having chronic disease history			640 (43.01)		2132 (40.26)
	Having past mental problem			145 (9.74)		622 (11.75)
**Frequency of face-to-face contact with friends before the pandemic, n (%)**						
	Once a month			986 (66.26)		3238 (61.15)
	2-3 times a month			238 (16)		1168 (22.06)
	Once a week			141 (9.47)		447 (8.44)
	≥2 times a week			123 (8.27)		442 (8.35)
**Remote communication with friends during the pandemic, n (%)**						
	**Having remote communication**			0 (0)		5295 (100)
		Voice calling^a^	0 (0)		3368 (63.61)	
		Text messaging^a^	0 (0)		5016 (94.73)	
		Video calling^a^	0 (0)		1135 (21.43)	
**Remote communication with friends before the pandemic, n (%)**						
	**Having remote communication**			772 (51.88)		5200 (98.2)
		Voice calling^a^	415 (27.89)		3733 (70.5)	
		Text messaging^a^	637 (42.81)		4934 (93.18)	
		Video calling^a^	102 (6.85)		842 (15.9)	
Loneliness, n (%)				311 (20.9)		1009 (19.05)

^a^We defined people who used each remote communication method once a month or more as using each type of communication.

**Table 3:** Association between using remote communication and loneliness during the COVID-19 pandemic by age^a,b^.

Variables				Aged <65 years									Aged ≥65 years									*P* value for interaction	
				Lonely, n/N (%)		Adjusted prevalence (%)		Adjusted PR^c^		95% CI		Lonely, n/N (%)			Adjusted prevalence (%)		Adjusted PR		95% CI				
**Family**																							
	**Remote communication with family members living apart**																						.47
		No	194/739 (26.3)		25.5		1		Ref^d^		31/264 (11.7)			10.8		1		Ref					
		Yes	564/2426 (23.2)		23.5		0.92		0.76-1.11		83/1054 (7.9)			8.1		0.75		0.44-1.28					
	**Voice calling with family members living apart**																						.03
		No	328/1288 (25.5)		25		1		Ref		55/443 (12.4)			11.8		1		Ref					
		Yes	430/1877 (22.9)		23.2		0.93		0.81-1.05		59/875 (6.7)			6.9		0.59		0.40-0.85					
	**Text messaging with family members living apart**																						.38
		No	245/910 (26.9)		27.2		1		Ref		46/423 (10.9)			10.5		1		Ref					
		Yes	513/2255 (22.7)		22.7		0.83		0.69-1.01		68/895 (7.6)			7.7		0.74		0.47-1.16					
	**Video calling with family members living apart**																						.14
		No	568/2333 (24.3)		25		1		Ref		82/980 (8.4)			8.2		1		Ref					
		Yes	190/832 (22.8)		21.2		0.85		0.71-1.01		32/338 (9.5)			10.2		1.25		0.75-2.08					
**Friends**																							
	**Remote communication with friends**																						.18
		No	260/1014 (25.6)		27.1		1		Ref		51/474 (10.8)			11.4		1		Ref					
		Yes	901/3875 (23.3)		22.9		0.85		0.75-0.95		108/1420 (7.6)			7.5		0.65		0.46-0.94					
	**Voice calling with friends**																						.009
		No	634/2606 (24.3)		24.6		1		Ref		83/809 (10.3)			10.8		1		Ref					
		Yes	527/2283 (23.1)		22.8		0.92		0.84-1.01		76/1085 (7)			6.7		0.62		0.47-0.82					
	**Text messaging with friends**																						.17
		No	278/1110 (25)		27.3		1		Ref		77/657 (11.7)			11		1		Ref					
		Yes	883/3779 (23.4)		22.8		0.84		0.75-0.93		82/1237 (6.6)			6.9		0.63		0.42-0.93					
	**Video calling with friends**																						.27
		No	919/3966 (23.2)		24.1		1		Ref		140/1682 (8.3)			8.1		1		Ref					
		Yes	242/923 (26.2)		22.4		0.93		0.83-1.03		19/212 (9)			11.2		1.38		0.69-2.75					

^a^Adjusted for age (singular and squared terms), gender, income, education level, work status, marital status, number of household members, chronic disease, past mental problems, frequency of face-to-face contact before the COVID-19 pandemic, and frequency of exposure variables before the COVID-19 pandemic.

^b^We defined people who used each remote communication method once a month or more as using each type of communication and categorized in “Yes.”

^c^PR: prevalence ratio.

^d^Ref: reference.

Across each communication tool, voice calling with family members living apart or friends was associated with a low prevalence of loneliness only among those aged ≥65 years, showing a between-group difference (family—aged <65 years: aPR=0.93, 95% CI 0.81-1.05; *P*=.24 vs aged ≥65 years: aPR=0.59, 95% CI 0.40-0.85; *P*=.005; *P*-for-interaction=.03 and friends—aged <65 years: aPR=0.92, 95% CI 0.84-1.01; *P*=.09 vs aged ≥65 years: aPR=0.62, 95% CI 0.47-0.82; *P*=.001; *P*-for-interaction=.009). Text messaging with friends was associated with a low prevalence of loneliness among both age groups (<65 years: aPR=0.84, 95% CI 0.75-0.93; *P*=.001 and ≥65 years: aPR=0.63, 95% CI 0.42-0.93; *P*=.02; *P*-for-interaction=.17). We observed qualitatively similar findings in the sensitivity analysis (Table S3 in Multimedia Appendix 1).

When stratified by gender, men had a stronger association between having remote communication with friends and a lower prevalence of loneliness than women (Table 4).

However, the statistical difference between the gender group was not apparent (men: aPR=0.75, 95% CI 0.63-0.89; *P*=.001 vs women: aPR=0.87, 95% CI 0.75-1.02; *P*=.08; *P*-for-interaction=.20).

Across each communication tool, all 3 types of remote communication with friends were associated with a low prevalence of loneliness for men (voice calling: aPR=0.78, 95% CI 0.66-0.92; *P*=.003; text messaging: aPR=0.75, 95% CI 0.64-0.89; *P*=.001; and video calling: aPR=0.82, 95% CI 0.71-0.96; *P*=.01), whereas only text messaging with friends was associated with a low prevalence of loneliness for women (voice calling: aPR=0.94, 95% CI 0.80-1.11; *P*=.45; text messaging: aPR=0.85, 95% CI 0.72-0.99; *P*=.04; and video calling: aPR=1.04, 95% CI 0.90-1.20; *P*=.60).

**Table 4:** Association between using remote communication and loneliness during the COVID-19 pandemic by gender^a,b^.

Variables				Men									Women									*P* value for interaction	
				Lonely, n/N (%)		Adjusted prevalence (%)		Adjusted PR^c^		95% CI		Lonely, n/N (%)			Adjusted prevalence (%)		Adjusted PR		95% CI				
**Family**																							
	**Remote communication with family members living apart**																						.46
		No	128/607 (21.1)		19.8		1		Ref^d^		97/396 (24.5)			21.8		1		Ref					
		Yes	238/1462 (16.3)		16.7		0.85		0.70-1.02		409/2018 (20.3)			20.8		0.95		0.74-1.22					
	**Voice calling with family members living apart**																						.41
		No	187/932 (20.1)		19.5		1		Ref		196/799 (24.5)			22.1		1		Ref					
		Yes	179/1137 (15.7)		16.1		0.83		0.71-0.96		310/1615 (19.2)			20.3		0.92		0.75-1.13					
	**Text messaging with family members living apart**																						.71
		No	160/789 (20.3)		19.4		1		Ref		131/544 (24.1)			24.8		1		Ref					
		Yes	206/1280 (16.1)		16.5		0.85		0.69-1.05		375/1870 (20.1)			19.9		0.80		0.63-1.02					
	**Video calling with family members living apart**																						.19
		No	291/1618 (18)		18.9		1		Ref		359/1695 (21.2)			21.3		1		Ref					
		Yes	75/451 (16.6)		14.3		0.76		0.56-1.01		147/719 (20.4)			20.2		0.95		0.80-1.13					
**Friends**																							
	**Remote communication with friends**																						.20
		No	169/854 (19.8)		21.7		1		Ref		142/634 (22.4)			23.5		1		Ref					
		Yes	372/2215 (16.8)		16.2		0.75		0.63-0.89		637/3080 (20.7)			20.5		0.87		0.75-1.02					
	**Voice calling with friends**																						.12
		No	296/1571 (18.8)		19.9		1		Ref		421/1844 (22.8)			21.6		1		Ref					
		Yes	245/1498 (16.4)		15.5		0.78		0.66-0.92		358/1870 (19.1)			20.3		0.94		0.80-1.11					
	**Text messaging with friends**																						.32
		No	188/996 (18.9)		21.4		1		Ref		167/771 (21.7)			24		1		Ref					
		Yes	353/2073 (17.0)		16.1		0.75		0.64-0.89		612/2943 (20.8)			20.3		0.85		0.72-0.99					
	**Video calling with friends**																						.03
		No	440/2539 (17.3)		18.3		1		Ref		619/3109 (19.9)			20.8		1		Ref					
		Yes	101/530 (19.1)		15.1		0.82		0.71-0.96		160/605 (26.4)			21.6		1.04		0.90-1.20					

^a^Adjusted for age (singular and squared terms), gender, income, education level, work status, marital status, number of household members, chronic disease, past mental problems, frequency of face-to-face contact before the COVID-19 pandemic, and frequency of exposure variables before the COVID-19 pandemic.

^b^We defined people who used each remote communication method once a month or more as using each type of communication and categorized as “Yes.”

^c^PR: prevalence ratio.

^d^Ref: reference.

## Discussion

### Principal Findings

In our study, among people who stopped face-to-face contact with friends during the COVID-19 pandemic, those who continued to contact them using remote communication technologies were 0.82 times less likely to experience loneliness than those who did not communicate using remote technologies. Among people who stopped face-to-face contact with family members living apart, having remote communication with them did not show an apparent association with loneliness. However, having a voice call and text messaging with family members living apart, as well as with friends, was associated with a low prevalence of loneliness. This association was stronger for text messaging than for voice calls. In contrast, video calling was not associated with low loneliness in both cohorts. When stratified by age, the association between voice calling with family members living apart or friends and loneliness was stronger among those aged ≥65 years than among those aged <65 years. Text messaging with friends was associated with low loneliness regardless of age. When stratified by gender, we did not find a difference in the association by type of remote communication with family members living apart or friends among men, but the association was only found in text messaging with friends among women.

To our knowledge, this is the first study to focus on the population who stopped meeting with family or friends during the pandemic, showing the association between technology-based remote communication and less loneliness. A recent study investigating the association between interhousehold contact and loneliness among older adults in the United States and United Kingdom in June 2020 reported that virtual contact had few mental health benefits [16]. Another study conducted in the United States in April 2020 reported that remote contact was not protective against loneliness [17]. However, these studies did not assess the change in face-to-face contact frequency during the pandemic, an unprecedented time that might increase loneliness [7]. A study investigating older Europeans during the pandemic suggested that people who had frequent face-to-face social contact might be at risk of increased loneliness because of physical distancing [33]. We focused on the participants who stopped meeting, which can be a risk factor for increasing loneliness, resulting in different findings from other studies that did not consider the change of face-to-face contact. In addition, an abovementioned study investigating US and UK data assessed the marginalized effect of the frequency increase of any remote communication, neither comparing the presence or absence of remote communication nor analyzing by tools. These differences may have led to distinct results. Given that feeling connectedness with intimate people, such as family and close friends, plays an essential role in mitigating loneliness [11], our findings indicate that having technology-based remote communication, especially via voice calling and text messaging, potentially provides such connectedness for people in situations where physical interaction is limited.

Notably, we found a strong relationship between text messaging with friends and a lower prevalence of loneliness. A study investigating pairs with strong ties revealed that they considered that they could communicate more constantly through text messaging than through voice calling because of their asynchronicity [12]. They also viewed text messaging as more private and direct than voice calling and communicated through text, aiming to maintain their relationship. Another recent study revealed that if couples were in a long-distance relationship, frequent text-based messaging was associated with high relationship satisfaction rather than voice or video calling [34]. Our results support and advance these findings: text-based communication with intimate others may provide a greater sense of connectedness and relationship satisfaction than calling, resulting in the prevention of loneliness when people cannot meet others. Technological advances, such as easy-to-use mobile devices and chat apps, have made text-based communication more convenient with intimate others. Designing social interventions based on the findings combined with technologies may be worth attempting to reduce loneliness.

Our findings from the stratified analysis highlight the importance of considering the sociodemographic status and selecting the preferred communication channels when constructing interventions to alleviate loneliness in the target population. For example, we found that those aged ≥65 years showed a lower prevalence of loneliness when using voice calling with family members living apart or friends compared with those aged <65 years. A previous study investigating the age difference in cell phone use in 2012 found that older adults preferred voice calling to contact friends and romantic partners more than younger adults [35]. Older people in health care settings also tended to favor telephone calls to communicate with relatives during the COVID-19 era [36]. Some studies focusing on older adults revealed an association between voice calling and low levels of loneliness [37,38]. In this context, experience and familiarity with remote tools may influence the effects of remote communication on loneliness, particularly among older adults.

Women had a weaker association between having remote communication and low loneliness than men, except for text messaging with friends. Sherman et al [39] revealed that women tend to bond more during in-person interactions than during video chat or instant messaging. A previous review of social ties and mental health indicated that women tend to mobilize more social support during periods of stress than men [40]. From these perspectives, men could get enough feeling of connectedness from any remote communication channel, whereas women may require various types of social relationships with in-person contact in stressful settings. Women’s hopes for social relationships might not have been fulfilled during the pandemic with social restrictions, resulting in a weaker association between remote communication and low loneliness. Additional support beyond remote communication with family members living apart or with friends may be needed for women to alleviate their loneliness under high social restrictions.

Our study has several limitations. First, because our study was cross-sectional, reverse causation is possible against the explanation that remote communication can reduce loneliness. Second, selection bias might have existed because the intimacy or quality of the relationship with family or friends before the pandemic, which could be related to the use of remote communication tools, was unknown. People who had intimate friends could maintain contact with them remotely, whereas those who did not may have lost communication opportunities during the pandemic. The intimacy level of their relationships before the pandemic may have affected the results, although we considered the frequency of meetings and the use of remote communication with family or friends before the pandemic. Third, we had no data on the baseline levels of loneliness before the pandemic. Although we adjusted for several factors affecting the baseline level of loneliness, our findings might be influenced by residual confounding factors. Detailed information about whom people communicated with and why they communicated was lacking. Fourth, the generalizability was limited because of the study conditions. We could focus on the population that stopped meeting with family or friends because of the COVID-19 pandemic; however, it is unknown whether our findings can be generalized outside the pandemic context. The interpretation of our results is also limited to people who can access the internet. In addition, given that there are cultural differences in the predictors of loneliness [41], the association between having remote communication and loneliness in other countries may be different from that in Japan. Fifth, we could not sufficiently consider and adjust the interactions between the types of remote communication devices. Text messaging with friends might affect the use of video calls with friends, and the reverse situation is also possible. Consequently, we could not completely distinguish the effects of one remote communication tool from those of the others. Further research with a longitudinal design and more information related to loneliness and remote communication is required.

### Conclusions

Our study showed that having remote communication, especially via voice calling and text messaging, was associated with low loneliness. Our findings also highlighted that the extent of the relationship between remote communication and loneliness could vary by age and gender. However, this association was especially prominent for text messaging with friends regardless of age and gender. These findings indicate that promoting and supporting such remote communication may help people who have limited access to face-to-face communication and experience loneliness. Longitudinal studies with detailed information at baseline are warranted to establish the causal relationships between remote communication and loneliness. On the basis of new findings, remote technologies can contribute to establishing an innovative approach to reduce loneliness during and after the COVID-19 pandemic.

## Appendix

Multimedia Appendix 1 Variables and questions for the frequency of communication, characteristic differences, sensitivity analysis, and correlation matrix of exposure variables.

## Abbreviations

aPR: adjusted prevalence ratio
JACSIS: Japan COVID-19 and Society Internet Survey
UCLA: University of California, Los Angeles

## Data Availability

Data are available upon reasonable request. The Japan COVID-19 and Society Internet Survey study data set is available upon request to the corresponding author.
