# Supplementary material for: Remote Communication and Loneliness During the COVID-19 Pandemic: Cross-Sectional Study
Source: J Med Internet Res. 2023 Jul 11;25:e45338. doi: 10.2196/45338 (PMC10369164; doi:10.2196/45338)
Supplement: Multimedia Appendix 1 [file jmir_v25i1e45338_app1.docx]

**Title: Remote Communication and Loneliness During the COVID-19 Pandemic: Cross-Sectional Study**

**Supplementary Tables**

**Table S1.** A list of variables and questionnaires for frequencies of communications

**Table S2.** Characteristic differences between the study cohort and the people who had face-to-face contact with family members living apart or friends during the COVID-19 pandemic in the JACSIS 2020 study.

**Table S3.** Sensitivity analysis: Association between using remote communication and loneliness during the COVID-19 pandemic by different age cut-off

**Table S4.** Correlation matrix of exposure variables.

Table S1: A list of variables and questionnaires for frequencies of communications

| Variables | Questions | Options |
| --- | --- | --- |
| The frequency of face-to-face communication with family members living apart before the COVID-19 pandemic | “How often did you see your families or relatives who are living apart before the COVID-19 pandemic (before January 2020)?” | “almost every day (6–7 times a week)”, “4–5 times a week”, “2–3 times a week”, “once a week”, “2–3 times a month”, “once a month”, and “not at all” |
| The frequency of face-to-face communication with friends before the COVID-19 pandemic | “How often did you see your friends before the COVID-19 pandemic (before January 2020)?” |  |
| The frequency of face-to-face communication with family members living apart during the COVID-19 pandemic | “How often did you see your families or relatives who are living apart in the past month (August 2020)?” |  |
| The frequency of face-to-face communication with friends during the COVID-19 pandemic | “How often did you see your friends in the past month (August 2020)?” |  |
| The frequency of voice calling with family members living apart before the COVID-19 pandemic | “How often did you make contact with your families or relatives who are living apart by voice call (e.g., telephone, mobile, LINE, Facebook Messenger, Skype) before the COVID-19 pandemic?” |  |
| The frequency of voice calling with friends before the COVID-19 pandemic | “How often did you make contact with your friends or neighbors by voice call (e.g., telephone, mobile, LINE, Facebook Messenger, Skype) before the COVID-19 pandemic?” |  |
| The frequency of text messaging with family members living apart before the COVID-19 pandemic | “How often did you make contact with your families or relatives who are living apart by email or text message (e.g., mobile, LINE, Facebook Messenger) before the COVID-19 pandemic?” |  |
| The frequency of text messaging with friends before the COVID-19 pandemic | “How often did you make contact with your friends by email or text message (e.g., mobile, LINE, Facebook Messenger) before the COVID-19 pandemic?” |  |
| The frequency of video calling with family members living apart before the COVID-19 pandemic | “How often did you make contact with your families or relatives who are living apart by video call (e.g., LINE, Facebook Messenger, Skype, Zoom) before the COVID-19 pandemic?” |  |
| The frequency of video calling with friends before the COVID-19 pandemic | “How often did you make contact with your friends by video call (e.g., LINE, Facebook Messenger, Skype, Zoom) before the COVID-19 pandemic?” |  |
| The frequency of voice calling with family members living apart during the COVID-19 pandemic | “How often did you make contact with your families or relatives who are living apart by voice call (e.g., telephone, mobile, LINE, Facebook Messenger, Skype) during the COVID-19 pandemic?” |  |
| The frequency of voice calling with friends during the COVID-19 pandemic | “How often did you make contact with your friends or neighbors by voice call (e.g., telephone, mobile, LINE, Facebook Messenger, Skype) during the COVID-19 pandemic?” |  |
| The frequency of text messaging with family members living apart during the COVID-19 pandemic | “How often did you make contact with your families or relatives who are living apart by email or text message (e.g., mobile, LINE, Facebook Messenger) during the COVID-19 pandemic?” |  |
| The frequency of text messaging with friends during the COVID-19 pandemic | “How often did you make contact with your friends by email or text message (e.g., mobile, LINE, Facebook Messenger) during the COVID-19 pandemic?” |  |
| The frequency of video calling with family members living apart during the COVID-19 pandemic | “How often did you make contact with your families or relatives who are living apart by video call (e.g., LINE, Facebook Messenger, Skype, Zoom) during the COVID-19 pandemic?” |  |
| The frequency of video calling with friends during the COVID-19 pandemic | “How often did you make contact with your friends by video call (e.g., LINE, Facebook Messenger, Skype, Zoom) during the COVID-19 pandemic?” |  |

Table S2. Characteristic differences between the study cohort and the people who had face-to-face contact with family members living apart or friends during the COVID-19 pandemic in the JACSIS 2020 study.

|  | | | Having face-to-face communication with family members living apart before the pandemic | | | | Having face-to-face communication with friends before the pandemic | | | |
| --- | --- | --- | --- | --- | --- | --- | --- | --- | --- | --- |
|  | | | No face-to-face communication with family members living apart during the pandemic (n = 4483, cohort 1) | | Having face-to-face communication with family members living apart during the pandemic (n = 9643) | | No face-to-face communication with friends during the pandemic (n = 6783, cohort 2) | | Having face-to-face communication with friends during the pandemic (n = 8680) | |
| Variables | | | n | % | n | % | n | % | n | % |
| Participants, No. | | | 4483 | 100 | 9643 | 100 | 6783 | 100 | 8680 | 100 |
| Demographic factors | | |  |  |  |  |  |  |  |  |
| Age, mean (SD), y | | | 52.3 | (16.2) | 50.2 | (16.2) | 52.2 | (15.7) | 48.7 | (17.4) |
| Sex | | |  |  |  |  |  |  |  |  |
|  | Male | | 2069 | 46.2 | 4407 | 45.7 | 3069 | 45.2 | 4155 | 47.9 |
|  | Female | |  |  |  |  |  |  |  |  |
| Income | | |  |  |  |  |  |  |  |  |
|  | Low (< 250 million yen) | | 1153 | 25.7 | 2513 | 26.1 | 1666 | 24.6 | 2139 | 24.6 |
|  | Intermediate (250 - 430 million yen) | | 1236 | 27.6 | 2843 | 29.5 | 1882 | 27.7 | 2558 | 29.5 |
|  | High (> 430 million yen) | | 1245 | 27.8 | 2685 | 27.8 | 1921 | 28.3 | 2554 | 29.4 |
|  | Refusal to answer | | 470 | 10.5 | 859 | 8.9 | 712 | 10.5 | 715 | 8.2 |
|  | Unknown | | 379 | 8.5 | 743 | 7.7 | 602 | 8.9 | 714 | 8.2 |
| Education | | |  |  |  |  |  |  |  |  |
|  | ≤12 years | | 1184 | 26.4 | 2738 | 28.4 | 1734 | 25.6 | 2367 | 27.3 |
|  | >12 years | | 3299 | 73.6 | 6905 | 71.6 | 5049 | 74.4 | 6313 | 72.7 |
| Employment status | | |  |  |  |  |  |  |  |  |
|  | Working | | 2684 | 59.9 | 6175 | 64.0 | 4199 | 61.9 | 5706 | 65.7 |
|  | House keeping | | 937 | 20.9 | 1856 | 19.2 | 1376 | 20.3 | 1359 | 15.7 |
|  | Not working | | 727 | 16.2 | 1381 | 14.3 | 1071 | 15.8 | 1188 | 13.7 |
|  | Student | | 135 | 3.0 | 231 | 2.4 | 137 | 2.0 | 427 | 4.9 |
| Marital status | | |  |  |  |  |  |  |  |  |
|  | Married | | 3097 | 69.1 | 6788 | 70.4 | 4694 | 69.2 | 5115 | 58.9 |
|  | Single | | 938 | 20.9 | 1891 | 19.6 | 1449 | 21.4 | 2586 | 29.8 |
|  | Widowed/Divorced | | 448 | 10.0 | 964 | 10.0 | 640 | 9.4 | 979 | 11.3 |
| Household size | | |  |  |  |  |  |  |  |  |
|  | 1 | | 771 | 17.2 | 1644 | 17.0 | 1115 | 16.4 | 1987 | 22.9 |
|  | 2 | | 1756 | 39.2 | 3552 | 36.8 | 2638 | 38.9 | 2810 | 32.4 |
|  | 3+ | | 1956 | 43.6 | 4447 | 46.1 | 3030 | 44.7 | 3883 | 44.7 |
| Health related factors | | |  |  |  |  |  |  |  |  |
| Having chronic disease history | | | 1846 | 41.3 | 3855 | 40.0 | 2772 | 40.9 | 3434 | 39.6 |
| Having mental health problem | | | 506 | 11.3 | 1197 | 12.4 | 767 | 11.3 | 1047 | 12.1 |
| Frequency of face-to-face contact before pandemic | | | with family members living apart | | | | with friends | | | |
| Once a month | | | 3386 | 75.5 | 4243 | 44.0 | 4224 | 62.3 | 2772 | 31.9 |
| Two to three times a month | | | 597 | 13.3 | 2408 | 25.0 | 1406 | 20.7 | 2629 | 30.3 |
| Once a week | | | 266 | 5.9 | 1658 | 17.2 | 588 | 8.7 | 1565 | 18.0 |
| Two times a week or more | | | 234 | 5.2 | 1334 | 13.8 | 565 | 8.3 | 1714 | 19.7 |
| Having Remote communication ^a^ | | | with family members living apart | | | | with friends | | | |
|  | | During the pandemic | 3386 | 75.5 | 8644 | 89.6 | 5295 | 78.1 | 8039 | 92.6 |
|  | | Before the pandemic | 3836 | 85.6 | 8915 | 92.5 | 5972 | 88.0 | 8281 | 95.4 |

^a^ We defined people who used any remote communication method once a month or more as having remote communication.

Table S3. Sensitivity analysis: Association between using remote communication and loneliness during the COVID-19 pandemic by different age cut-off ^a,b^

|  | | < 60 years old | | | | | ≥ 60 years old | | | | | P for interaction |
| --- | --- | --- | --- | --- | --- | --- | --- | --- | --- | --- | --- | --- |
| Variables | | Lonely, No./Total No. | Crude prevalence | Adjusted prevalence | Adjusted PR | 95%CI | Lonely, No./Total No. | Crude prevalence | Adjusted prevalence | Adjusted PR | 95%CI |  |
| A. Family | |  |  |  |  |  |  |  |  |  |  |  |
| Remote communication with family members living apart | | | | | |  |  |  |  |  |  |  |
|  | No | 181/653 | 27.7% | 26.4% | 1 | ref | 44/350 | 12.6% | 12.8% | 1 | ref | 0.47 |
|  | Yes | 519/2079 | 25.0% | 25.4% | 0.96 | [0.79, 1.17] | 128/1401 | 9.1% | 9.1% | 0.71 | [0.48, 1.06] |  |
| Voice calling with family members living apart | | | | | |  |  |  |  |  |  |  |
|  | No | 306/1131 | 27.1% | 26.8% | 1 | ref | 77/600 | 12.8% | 12.0% | 1 | ref | 0.18 |
|  | Yes | 394/1601 | 24.6% | 24.8% | 0.92 | [0.81, 1.05] | 95/1151 | 8.3% | 8.5% | 0.71 | [0.50, 1.02] |  |
| Text messaging with family members living apart | | | | | |  |  |  |  |  |  |  |
|  | No | 222/788 | 28.2% | 27.4% | 1 | ref | 69/545 | 12.7% | 14.4% | 1 | ref | 0.02 |
|  | Yes | 478/1944 | 24.6% | 24.9% | 0.91 | [0.74, 1.11] | 103/1206 | 8.5% | 8.1% | 0.56 | [0.40, 0.79] |  |
| Video calling with family members living apart | | | | | |  |  |  |  |  |  |  |
|  | No | 524/2031 | 25.8% | 26.3% | 1 | ref | 126/1282 | 9.8% | 10.3% | 1 | ref | 0.84 |
|  | Yes | 176/701 | 25.1% | 23.7% | 0.90 | [0.74, 1.10] | 46/469 | 9.8% | 8.7% | 0.85 | [0.50, 1.44] |  |
| B. Friends | |  |  |  |  |  |  |  |  |  |  |  |
| Remote communication with friends | | | | | |  |  |  |  |  |  |  |
|  | No | 235/877 | 26.8% | 28.0% | 1 | ref | 76/611 | 12.4% | 14.0% | 1 | ref | 0.03 |
|  | Yes | 840/3390 | 24.8% | 24.5% | 0.87 | [0.76, 1.00] | 169/1905 | 8.9% | 8.6% | 0.61 | [0.46, 0.82] |  |
| Voice calling with friends | | | | | |  |  |  |  |  |  |  |
|  | No | 592/2309 | 25.6% | 26.1% | 1 | ref | 125/1106 | 11.3% | 11.8% | 1 | ref | 0.02 |
|  | Yes | 483/1958 | 24.7% | 24.2% | 0.92 | [0.85, 1.01] | 120/1410 | 8.5% | 8.3% | 0.70 | [0.57, 0.87] |  |
| Text messaging with friends | | | | | |  |  |  |  |  |  |  |
|  | No | 249/943 | 26.4% | 28.0% | 1 | ref | 106/824 | 12.9% | 14.1% | 1 | ref | 0.03 |
|  | Yes | 826/3324 | 24.8% | 24.5% | 0.87 | [0.77, 0.99] | 139/1692 | 8.2% | 7.9% | 0.56 | [0.38, 0.82] |  |
| Video calling with friends | | | | | |  |  |  |  |  |  |  |
|  | No | 845/3420 | 24.7% | 25.6% | 1 | ref | 214/2228 | 9.6% | 9.6% | 1 | ref | 0.53 |
|  | Yes | 230/847 | 27.2% | 23.9% | 0.93 | [0.84, 1.05] | 31/288 | 10.8% | 10.7% | 1.11 | [0.66, 1.87] |  |

^a^ Adjusted for age (singular and squared terms), gender, income, education level, work status, marital status, household number, chronic disease, past mental history, frequency of face-to-face contact before the COVID-19 pandemic, and frequency of exposure variables before the COVID-19 pandemic.

^b^ We defined people who use each remote communication method once a month or more as using each type of communication, “Yes.”

Table S4. Correlation matrix of exposure variables.

1. Correlations of voice calling, text messaging, and video calling with family members living apart among the participants who stopped meeting with family members living apart.

| Communication with family members living apart | Voice calling | Text messaging | Video calling |
| --- | --- | --- | --- |
| Voice calling | 1.00 | - | - |
| Text messaging | 0.52 | 1.00 | - |
| Video calling | 0.36 | 0.30 | 1.00 |

2. Correlations of voice calling, text messaging, and video calling with family members living apart among the participants who stopped meeting with friends.

| Communication with family members living apart | Voice calling | Text messaging | Video calling |
| --- | --- | --- | --- |
| Voice calling | 1.00 | - | - |
| Text messaging | 0.41 | 1.00 | - |
| Video calling | 0.34 | 0.23 | 1.00 |
